# On the Performance of Video Quality Assessment Metrics under Different Compression and Packet Loss Scenarios

**DOI:** 10.1155/2014/743604

**Published:** 2014-05-20

**Authors:** Miguel O. Martínez-Rach, Pablo Piñol, Otoniel M. López, Manuel Perez Malumbres, José Oliver, Carlos Tavares Calafate

**Affiliations:** ^1^Department of Physics and Computer Engineering, the Miguel Hernández University, Avenida de Universidad s/n, Elche, 03202 Alicante, Spain; ^2^Department of Computer Engineering, Polytechnic University of Valencia, Camino de Vera s/n, Building G1, 46022 Valencia, Spain

## Abstract

When comparing the performance of video coding approaches, evaluating different commercial video encoders, or measuring the perceived video quality in a wireless environment, Rate/distortion analysis is commonly used, where distortion is usually measured in terms of PSNR values. However, PSNR does not always capture the distortion perceived by a human being. As a consequence, significant efforts have focused on defining an objective video quality metric that is able to assess quality in the same way as a human does. We perform a study of some available objective quality assessment metrics in order to evaluate their behavior in two different scenarios. First, we deal with video sequences compressed by different encoders at different bitrates in order to properly measure the video quality degradation associated with the encoding system. In addition, we evaluate the behavior of the quality metrics when measuring video distortions produced by packet losses in mobile ad hoc network scenarios with variable degrees of network congestion and node mobility. Our purpose is to determine if the analyzed metrics can replace the PSNR while comparing, designing, and evaluating video codec proposals, and, in particular, under video delivery scenarios characterized by bursty and frequent packet losses, such as wireless multihop environments.

## 1. Introduction

In the past years, the development of novel video coding technologies has spurred the interest in developing digital video communications, where evaluation mechanisms to assess the video quality play a major role in the overall design of video communication systems.

The most reliable way of assessing the quality of a video is subjective evaluation, because human beings are the ultimate receivers in most applications. The mean opinion score (MOS), which is a subjective quality metric obtained from a number of human observers, has been regarded for many years as the most reliable form of quality measurement. However, the MOS method is too cumbersome, slow, and expensive for most applications. Objective quality assessment metrics (QAM) are valuable because they provide video designers and standard organizations with means for making meaningful quality evaluations without convening viewer panels.

Recently, new objective image and video quality metrics have been proposed. They emulate human perception of video quality since they produce results which are very similar to those obtained from subjective methods. Most of these proposals were tested and compared in the different phases carried out by the video quality experts group (VQEG), which was formed to develop, validate, and standardize new objective measurement and comparison methods for video quality. The models that the VQEG forum validates result in International Telecommunication Union (ITU) recommendations and standards for objective quality measurement for both television and multimedia applications [1]. Some of the QAM proposals are designed to be as generalist as possible, that is, to be able to assess quality for a wide set of different distortion types, while other QAM focus their design on the detection of one, two, or a reduced set of specific distortions.

It would be desirable to find a QAM for image and video that exhibits a good behavior for any set of video and/or image distortions, that is, that detects accurately (as close as possible to the human perceived quality) any distortion regardless of its type and degree. Also, it would be desirable that the time required to obtain a quality measurement is short enough in order to have a practical use or even to be able to use it in real time.

But quality is by definition a highly subjective feature that is influenced not only by the intrinsic characteristics of the signal but also by psychological and environmental factors. Therefore, the task of choosing “the best QAM” is influenced by too many factors and sources of inaccuracy. These sources of inaccuracy are, for example, the reliability of unbiased subjective reference data, the selection of video or image contents, the degree of the impairments and where they appear (in space and time), the procedure used to map between subjective and objective quality values, and even the use and interpretation of the correlation indicators. These factors must be taken into account when making comparisons between metrics [2].

The selection of a QAM may also depend on the target application where it will be used. Examples of applications are a real-time monitor that adaptively adjusts the image quality in a video acquisition or transmission system, a benchmarking image processing system, or even algorithms and encoder proposals that are embedded into image processing systems to decide about the preprocessing and postprocessing stages.

We work with a set of the most relevant quality assessment metrics whose source code or test software has been made available by their authors. So, we can use them in our own evaluation tests.

As mentioned before, we will analyze the behavior of the candidate metrics in two test environments. The first one, is the compression environment, where the quality of compressed sequences at different bitrates with different encoders is compared by means of QAM. The most common way of doing the comparisons between existing image/video coding approaches, improvements over these approaches, or completely new codec designs is by performing a rate/distortion (R/D) analysis. When using R/D, the distortion is usually measured in terms of PSNR (peak signal-to-noise ratio) values, where rates are often measured in terms of bpp (bits per pixel) for images or bps (bits per second) for video. So, in this test environment, we work with the selected QAM as candidates to replace the PSNR as the distortion metric in the R/D comparisons. We will also consider the QAM complexity in order to determine their applicability. The second one is the packet loss environment, where we will analyze the behavior of the candidate metrics in the presence of packet losses under different mobile ad hoc networks (MANET) scenarios. In particular, we are going to compare the behavior of QAM when measuring the quality degradation of an H.264/AVC video delivery in a MANET. We use a hidden Markov model (HMM) to accurately reproduce the packet loss patterns typical of these networks, including variable network congestion levels and different degrees of node mobility. For each particular network scenario, we perform a bitstream erasure process based on the loss patterns suggested by the HMM model. The resulting bitstream is delivered to the H.264/AVC decoder in order to get the resulting HRC that will be used to calculate the QAM value.

The organization of the paper is as follows. In the next section, we will describe the main frameworks addressing objective QAM. In Section 3, we will expose some key aspects of how to compare heterogeneous metrics and the method used to compare the metrics under evaluation. In Section 4, we show the behavior of several available quality metrics in the compression environment. In Section 5, the models and the methods used for the packet loss environment are explained and a behavioral analysis of the metrics is made for different network scenarios. Finally, in Section 6, we present the main conclusions of this work.

## 2. Objective Quality Assessment Metrics

In the past years, a big effort has been done in the field of QAM. A large number or objective metrics can be found in the literature. Some of them have been designed for a specific kind of distortions, while others are more generalist and try to assess quality regardless of the distortion type. Besides, each metric design is different. Objective evaluation of picture quality in line with human perception is still difficult [3–9] due to the complex, multidisciplinary nature of the problem, including aspects related to physiology, psychology, vision research, and computer science. Nevertheless, with proper modeling of major underlying physiological and psychological phenomena and by obtaining results from psychophysical tests and experiments, it is possible to develop better visual quality metrics to replace nonperceptual criteria as PSNR or MSE being still widely used nowadays.

In the literature, we can find different classifications and frameworks that group several QAM depending on the way they are designed. In this section, we will briefly describe the main ideas behind the different frameworks, along with their main QAM.

There is a consensus in a primer classification of objective quality metrics [10, 11] attending to the availability of original nondistorted info (video reference) to measure the quality degradation of available distorted versions.

- Full reference (FR) metrics perform the distortion measure with full access to the original image/video version, which is taken as a perfect reference.
- No reference (NR) metrics have no access to the reference image/video. So, they have to perform the distortion estimation based on the distorted version only. In general they have lower complexity but are less accurate than FR metrics and are designed for a limited set of distortions and video formats.
- Reduced reference (RR) metrics have access to partial information about the original video. A RR metric defines what information has to be extracted from original video, so it can be compared with the the same one extracted from the distorted version.

**Figure 1:**
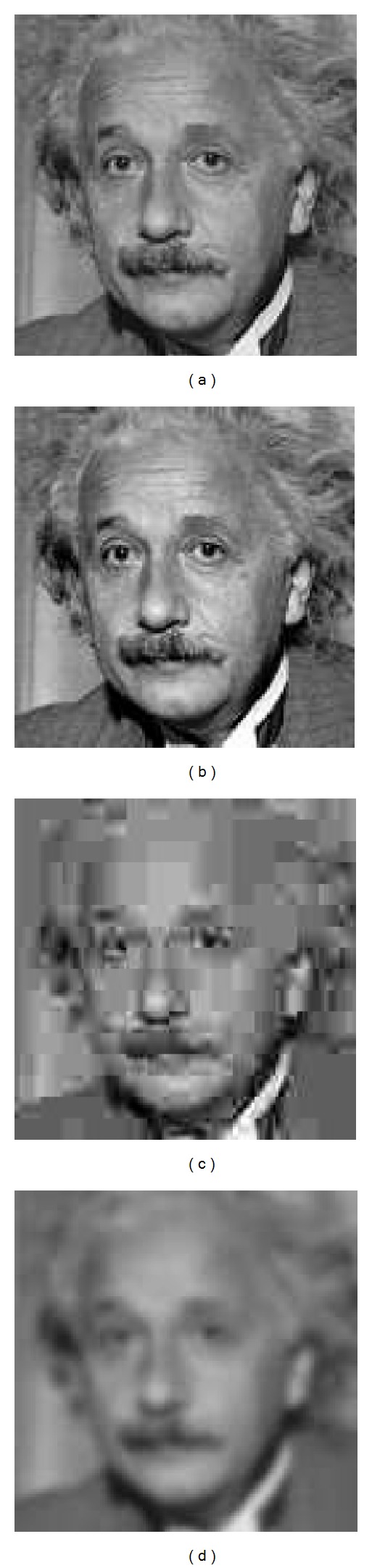
Example of three figures with different impairments and the same PSNR values: (a) original, (b) contrast stretched 26.55 dB, (c) JPEG compressed 26.60 dB, and (d) blurred 26.55 dB.

The most widely used FR objective video quality metrics are the mean square error (MSE) and the peak signal-to-noise ratio (PSNR). They are simple and quick to calculate, providing a good way to evaluate the video quality [12]. However, it is well known that these metrics do not always capture the distortion perceived by the human visual system (HVS). In Figure 1, an original image has been distorted in different ways. The PSNR metric gives almost the same value for each distortion, indicating that the quality of the distorted images is the same, but as it can be seen, the perceived quality is different for each image. Moreover, it is not unusual that the perceived quality of image in Figure 1(b) is higher than the one given to the original one, Figure 1(a). That is, a distorted image has better perceptual quality than the original one. If PSNR is used for measuring the quality of the resulting images/videos produced by the different coding proposals, how can we certify that one coding proposal has a better perceptual quality than another?

In this section, we will briefly describe also the main ideas behind the different frameworks and the most relevant and cited QAM of each one. QAM can be classified by many factors such as the metric architecture (number and type of blocks and stages or algorithms used in the metric design), the primary domain (space or frequency) where they work, and the inclusion or not of HVS characteristics or HVS models in their design.

### 2.1. HVS Model Based Framework

A basic idea of any metric based on a HVS model is that subjective differences between two images cannot be extracted directly from the given images (original and distorted one) but from their perceived versions, that is, from the version that our brain perceives. As it is known, the HVS produces several visual scene information reductions, carried out in different steps. The way in which this information reduction process is modeled is the key to obtain a good subjective fidelity metric.

This framework includes the metrics that are clearly based on a HVS model, that is, their design follow the stages of any of the available HVS models. We include here metrics from the error sensitivity framework (ESF) [7] and also some other RR and NR metrics that are based on HVS models.

This framework mainly include FR metrics based on HVS models that measure errors between the reference and the distorted content using a HVS model.

In general, the emulation of HVS is a bottom-up approach that follows the first retina processing stages to continue with different models of the visual cortex behavior. Also, some metrics deal with cognitive issues about the human visual processing modeling that are included as additional stages.

The main difference between the FR metrics of this framework is related to the way they perform the subband decomposition inspired by the complex HVS models [13–15], low cost decompositions in DCT [16, 17] or wavelet [18] domains, and with other HVS related issues like in [19] where foveal vision is also taken into account and in [20] where focus of attention is also considered. It is worth noting that most of proposed FR quality assessment models share the error sensitivity based philosophy which is motivated from psychophysical vision science research [11].

**Figure 2:**
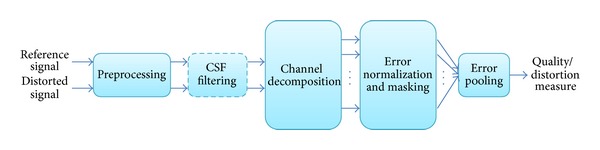
Common block diagram of the error sensitivity framework.

Figure 2 shows a block diagram with the typical processing stages of a FR metric. In the preprocessing stage, different operations are done in order to adequate some characteristic of the reference and the distorted input versions. These operations commonly include pixel alignment, image cropping, color space transformations, device calibrations, PSF filtering, light adaptation, and other operations. Not all the metrics perform all these operations; each metric processes both signals in a different way. After the preprocessing stage, usually HVS models first decompose the input signal into spatiotemporal subbands at both the reference and distorted signals.

The contrast sensitivity function (CSF) can be implemented in the channel decomposition step by the use of linear filters that approximate the frequency responses to the CSF like in [21]. But most of the metrics choose to implement the CSF as weighting factors that are applied to the channels after the channel decomposition, providing for each channel a different perceptual sensitivity.

As mentioned before, frequency decomposition is one of the biggest differences between models and hence between metrics. Complex HVS frequency channel decomposition models are used in QAM designs, but some of these models are simplified attending to computational constraints. In this sense, other QAM use the DCT [16] or wavelet [18] transforms showing good MOS correlation results. Depending also on the metric type and the distortions it handles, metrics use different channel decomposition models.

Cortical receptive fields are represented by 2D Gabor functions, but the Gabor decomposition is hard to compute and is not suitable for some operations as invertibility, reconstruction by addition, and so forth. In [22], Watson modeled a frequency and orientation decomposition with profiles similar to the 2D Gabor functions but computationally more efficient. Other authors like Lubin [23], Daly [24], Teo and Heeger [13], and Simoncelli et al. [25] provided different models trying to approximate as close as possible the HVS channel decomposition.

There are also some models that use temporal frequency decomposition in order to account for the characteristics of the temporal mechanisms in the HVS [21, 26]. The design of temporal filter banks is typically implemented using infinite impulse response filters (IIR) with a delay of only a few frames; other authors use finite response filters that despite their higher delay are simpler to implement.

The next step is error normalization and masking. Masking occurs when a stimulus that is visible by itself cannot be detected due to the presence of another stimulus. In contrast, facilitation occurs when a nonvisible stimulus becomes visible due to the presence of another stimulus. Most of the HVS models implement error normalization and masking as a gain-control mechanism, using the contrast visibility thresholds to weight the error signal at each channel. Some metrics [14], due to complexity and performance issues, use only intrachannel masking, while others [13] include interchannel masking, as there are evidences that channels are not totally independent in the HVS. Other authors [27] include also in this stage the luminance masking, also called light adaptation. In [28, 29], some comparisons of different masking models and some considerations about how to include them into an image encoder are made. In [30], authors propose a contrast gain-control model of the HVS that incorporates also a contrast sensitivity function for multiple oriented bandpass channels.

The last processing step (Figure 2) is the error pooling, which is in charge of combining the error signals in different channels into a single distortion/quality interpretation, providing different importance to errors depending on the channels where they appear. For most QAM, a Lp norm or Minkowski norm is used to produce an image spatial error maps. From the spatial error map, a frame-level distortion score is computed. In video quality assessment, we obtain the corresponding sequence-level distortion score by averaging frame scores. For the time domain, some metrics use temporal HVS models or information to accurately reproduce human scores, while others simply do not assess time domain. Other QAM that may be included in the model based framework may be found in [13, 15–21, 26, 27, 31–36].

### 2.2. HVS Properties Framework

In this framework we consider the metrics that, although are not based on a specific HVS model, are still inspired in features of the HVS. We also include those metrics that are designed to detect specific impairments produced by any of the processing stages of image and video coding, like quantization, transmission errors, and so forth.

The Institute for Telecommunication Sciences (ITS) presented in [37] an objective video quality assessment system that was based on human perception. They extract several features from the original and degraded video sequences that were statistically analyzed in comparison with the corresponding human rating extracted form subjective tests. This analysis provide parameters to adjust objective measures for these features and, after being combined in a simple linear model, they provide the final predicted scores. Some of the extracted features require the presence of the original sequence, while others are extracted in a no-reference mode. The proposed metric exploits spatial and temporal information. The processing include Sobel filtering, Laplace filtering, fast Fourier transforms, first-order differencing, color distortion measures, and moment calculation.

In [38], authors proposed a RR metric for in-service quality monitoring system. Their metric is built on a set of spatiotemporal distortion metrics that can be used for monitoring in-service of any digital video system. Authors expose that a digital video quality metric, in order to be widely applicable, must accurately emulate subjective responses, must work over the full range of quality (from very low bit rate to very high), must be computationally efficient, and should work for end-to-end in-service quality monitoring. The metrics are based on extracted features from the video sequence as in [37] and in order to satisfy the last condition (to be able to work in in-service monitoring systems), these features, extracted from spatiotemporal regions are sent, compressed following the ITU-R Recommendation BT.601, through an ancillary data channel so that it can be continuously transmitted. In the paper, the authors describe these spatiotemporal distortion metrics in detail, so that they can be implemented by researchers.

Later, through The National Telecommunications and Information Administration (NTIA), the same authors, proposed the general model of the video quality measurements techniques (known as VQM metric [39, 40]) for estimating video quality and its associated calibration techniques. This metric was submitted to be independently evaluated on MPEG-2 and H.263 video systems by the video quality experts group (VQEG) in their phase II full reference television (FR-TV) test. In [41], authors reduce the requirements of some of the features extracted in the NTIA general model in order to achieve a monitoring system that uses less than 10 kbits/s of reference information.

We also can find metrics based on watermarking techniques that analyze the quality degradation of the embedded image [42]. There are metrics that are designed for measurement-specific distortions types and those produced by specific encoders [43, 44]. Another representative metrics in this framework are the ones proposed in [43–49].

### 2.3. Statistics of Natural Images Framework

Some drawbacks of the model based HVS framework are reviewed in [7, 50]. Some of these drawbacks are, for example, that the HVS models work appropriately for simple spatial patterns, like pure sine waves; however, when working with natural images, where several patterns coincide in the same image area, their performance degrades significantly. Another drawback is related to the Minkowski error pooling, as it is not a good choice for image quality measurement. As authors show, different error patterns can lead to the same final Minkowski error.

Therefore, several authors argue that the approach to the problem of perceptual quality measurement must be a top-down approach, analyzing the HVS to emulate it at a higher abstraction level. The authors supporting this approach propose using the statistics of the natural images. Some of them propose the use of image statistics to define the structural information of an image. When this structural information is degraded, then the perceptual quality is also degraded. In that sense, a measurement of the structural distortion should be a good approximation to the perceived image distortion. These metrics are able to distinguish between distortions that change the image structure and distortions that do not change it, like changes in luminance and contrast.

In [7, 51], authors define a Universal Quality Index that is able to determine the structural information of the scene. This index models any distortion as a combination of three different factors: (a) the loss of correlation between the original signal and the distorted one, (b) the mean distortion that measures how close the mean of the original and distorted version are, and (c) the variance distortion that measures how similar the variances of the signals are. The dynamic range of the Quality Index is [−1,1]. A value of 1 indicates that both signals are identical. They apply this index in a 8 × 8 window for an image, obtaining a quality map of the image. The overall index is the average of the quality map.

Authors in [50] further improve their previous quality index and in [52] propose a generalization of their work where any distortion may be decomposed into a linear combination of different distortion components. In [53], the model is extended to the complex wavelet domain in order to design a robust metric to scaling, rotation, and translation effects.

Authors in [54] proposed a video quality metric following a frame by frame basis. It takes quality measures for different blocks of each frame taking into account their spatial variability, the movement, and other effects (like blocking) by means of a specifically adapted NR metric [45].

Other authors use also statistics of the natural scene in a different way. They state that the statistical patterns of natural scenes have modulated the biological system, adapting the different HVS processing layers to these statistics. First a general model of the natural images statistics is proposed. The modeled statistics are those captured with high quality devices working in the visual spectrum (natural scenes). So, text images, computer generated graphics, animations, draws, random noise or image, and videos captured with nonvisual stimuli devices like radar, sonar, X-ray, and so forth are out of the scope of this approach. Then, for a specific image, the perceptual quality is measured taking into account how far its own statistics are from the modeled ones. In [55], a statistical model of a wavelet coefficient decomposition is proposed, and in [56] the authors propose an NR metric derived from previous work.

Some metrics defined under this approach take the objective quality assessment as an information loss problem, using techniques related to information theory [57, 58].

### 2.4. Metrics under Study

Now, we introduce the metrics we will use in our study. The criteria to choose these metrics, and no other ones, was the availability of their code (source or executable) to reproduce their behavior as follows.

- The DMOSp-PSNR metric: we translate the traditional PSNR to the DMOS space applying a scale-conversion process. We call the resulting metric DMOSp-PSNR.
- The Mean Structural SIMilarity index [50] (MSSIM) from the structural distortion/similarity framework: in the reference paper, this FR metric was tested against JPEG and JPEG2000 distortion types. We test its performance with the new distortion types available in the second release of Live Database, “Live2 Database” since it is considered a generalist metric.
- The visual information fidelity (VIF) metric [59] from the Statistics of Natural Images Framework. A FR metric that quantifies the information available in the reference image and determine how much of this reference information can be extracted from the distorted image.
- The no-reference JPEG2000 quality assessment (NRJPEG2000) [54] from the Statistics of Natural Images Framework. A NR metric that uses natural scene statistical models in the wavelet domain and uses the Kullback-Leibler distance between the marginal probability distributions of wavelet coefficients of the reference and distorted images as a measure of image distortion.
- Reduced-reference image quality assessment (RRIQA) [57] from the Statistics of Natural Images Framework. The only RR metric under study. It is based on a natural image statistical model in the wavelet transform domain.
- The no-reference JPEG quality score (NRJPEGQS) [43] from the HVS properties framework. A NR metric designed specifically for JPEG compressed images.
- The video quality metric [40] (VQM general model) from the HVS properties framework. The VQM uses RR parameters sent through an ancillary channel that requires at least 14% of the uncompressed sequence bandwidth. Although being conceptually an RR metric, it was submitted to the VQEG FR-TV test because the ancillary channel can be used to receive more detailed and complete references from the original frames, even the original frames themselves.

## 3. Comparing Heterogeneous Metrics

As previously mentioned, each QAM gets the quality of the image/video using its own and specific scale that depends on its design. Therefore, these raw quality scores cannot be compared directly, even though the range of the values (scale) is the same. In order to compare fairly the behavior of various metrics for a set of images or sequences, the objective quality index obtained from each metric has to be converted into a common scale.

**Figure 3:**
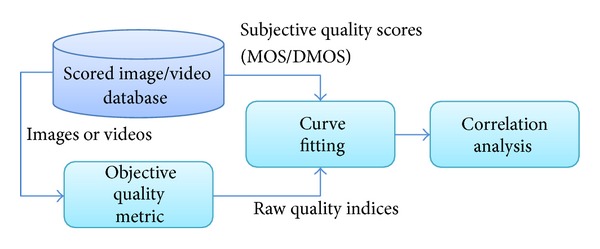
Block diagram of the QAM evaluation process.

When reviewing the performance comparisons that authors made in their new QAM proposals, few details are provided about the comparison procedure itself. So, it is difficult to replicate these results. Authors in [2] reviewed the sources of inaccuracy of each step of the QAM comparing process, shown at Figure 3. The sources of inaccuracy may be related to many factors as the reliability of the subjective reference data, the types and grade of the distortions in the images or videos, the selection of the content that made up the training and testing sets, and even the use and interpretation of the correlation indicators. These sources of inaccuracy can lead to quantitative differences when the same QAM is tested by different authors, even when the tests are correctly done. Although different tests can provide slightly varying results for a set of metrics, their results should be in line as explained in [2].

These issues encouraged and guided us to perform our own comparison test with the selected QAM in order to adapt the test to the target applications we are interested in. The results of our test, as expected, were slightly different from other comparison tests but remain in line with their results [2].

We use the method and mapping function proposed by the VQEG [6, 60] with some refinements proposed in other relevant comparison tests [61]. The chosen target scale is the DMOS scale (differences mean opinion score) which is the one used by the VQEG and other authors [61] when comparing metric proposals.

In order to compare several QAM, first a subjective test must be done, for example, a Double Stimulus Continuous Quality Scale (DSCQS) method as suggested and explained in [6], in order to get the subjective quality assessment of a set of images or sequences. The scale used by the viewers goes from 0 to 100. Raw scores obtained in subjective tests are converted into difference scores and processed further [58] to get a linear scale in the 0–100 range. The mean opinion score (MOS) can be calculated for the source and distorted versions of each image or sequence in this set. The DMOS is therefore the difference between the MOS value obtained for the original image/sequence and the MOS value obtained for the distorted one. So, for a particular image or sequence, its DMOS value gives the mean subjective value of the difference between the original and the distorted versions. A value of 0 means no subjective difference found between the images by all the viewers. Due to the nature of the subjective test this value is very unlikely.

In this work, we have not done such a subjective test. Instead of this, we have used directly the DMOS values published in the Live Database Release 2 [62] and in the VQEG Phase I Database [63].

Basically, the raw score of each metric must be converted into a value in this predicted DMOS (DMOSp) scale. This is done in the curve fitting step, shown in Figure 3. The final result of this scale-conversion process allows the quality score given by a metric for a specific image/sequence to be directly comparable with the one given by the other metrics for the same image/sequence.

We use the nonlinear mapping function between the objective and the subjective scores, as suggested in the VQEG Phase I and Phase II testing and validation tests [6, 60] as well as in other extensive metrics comparison tests [61]. This function is shown in (1). It is a parametric function which is able to translate a QAM raw score to the DMOSp space. As suggested in [2, 64], the performance evaluation of the metrics (correlation analysis step in Figure 3) is computed after a nonlinear curve fitting process.

A linear mapping function cannot be used because quality scores are rarely scaled uniformly in the DMOS scale, because different subjects may interpret vocabulary and intervals of the rating scale differently, depending on the language, viewing instructions, and individual psychological characteristics. Therefore, a linear mapping function would give too pessimistic view of the metric performance. Several mapping functions could be selected for this purpose, such as cubic, logistic, exponential, and power functions, with monotonicity being the main property that the function must comply with, at least in the relevant range of values.

Consider

(1) $$\begin{array}{c} \text{Quality}\left(x\right)=\beta_{1}\text{logistic}\left(\beta_{2},\left(x-\beta_{3}\right)\right)+\beta_{4}x+\beta_{5}, \end{array}$$

(2) $$\begin{array}{c} \text{logistic}\left(\tau ,x\right)=\frac{1}{2}-\frac{1}{1+\exp\left(\tau x\right)}. \end{array}$$

Equation (1) has five parameters, from *β*_1_ to *β*_5_, that are fixed by the curve fitting process that achieves the best correlation between the QA metric values and the subjective DMOS values. We have not found in the literature any mapping function with its parameters for any image/video database. So, we have calculated these parameters based on sets of images and sequences that conform with our “training sets”.

**Figure 4:**
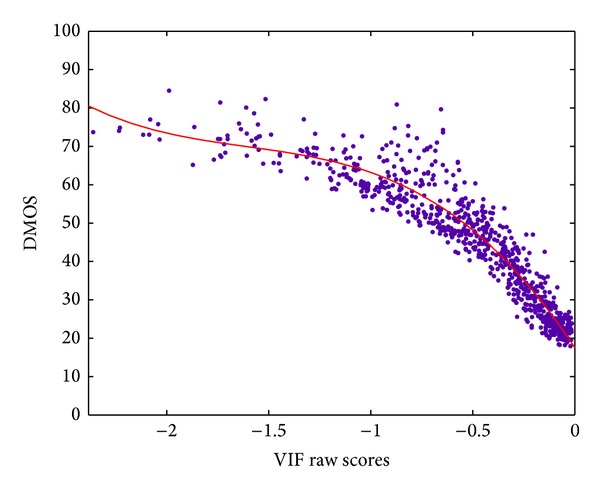
Dispersion plot used for the VIF metric including the curve fit for (1).

As an example, Figure 4 shows the dispersion plot used in the fitting process for one of the metrics, in this case the VIF metric. Each point of the scatter-plot corresponds to an image of the training set used, Live2 Database [62]. For each image in the training set, we get the average DMOS value obtained in the subjective test and we run each metric in order to get its raw quality scores. Each metric gives its score in its own scale.

The *x*-axis of Figure 4 corresponds to the raw values given by the VIF implementation used, where 0 corresponds to the highest quality reported by the metric and decreasing values report lower quality. In the *y*-axis, we have the corresponding DMOS values. The curve fitting process gives us the parameters for (1), which is represented by the solid curve in Figure 4.

The quality of the images in the subjective test is variable, covering a large range of distortion types and intensities for each distortion. Image distortions go from very highly distorted to practically undistorted ones. The viewers gave their scores for each image in the set, obtaining the average DMOS value. As shown in Figure 4, the dynamic range of the average DMOS values does not reach the limits of the DMOS scale (0 and 100) for any distortion type; therefore, the fitted curve predicts DMOSp values inside the same dynamic range. This is the reason why for a raw score of 0 (the best possible quality for the metric in this case), the predicted DMOSp value is not 0; that is, there was no image scored with an average DMOS value of 0, instead of that, the best DMOSp value obtained is around the value of 20. So, in the case of the VIF metric its dynamic DMOSp range varies from 20 to 80.

Having fixed the beta parameters for each metric (see Table 1), (1) can be used to estimate or predict the DMOSp value for any objective metric score.

In Table 2, the performance of our fittings is shown. These performance parameters show the degree of correlation between the DMOSp values and the subjective DMOS values provided by the viewers. Performance validation parameters are the Pearson correlation coefficient (PCC), the root mean squared error (RMSE), and the Spearman rank order correlation coefficient (SROCC).

Another key point to consider while comparing QAM [2] is the selection of the image or video sequence set used as “training set.” The “training set” is used to perform the curve fitting process. This set should be chosen with special care and must be excluded from validation tests. So for each metric, the fitting process must be done using images or sequences with impairments that the metric is designed to handle. See [2] for details of how an incorrect selection of the image “training set” can influence the final interpretation of the statistics used in the correlation analysis.

Once the metric has been evaluated in the correlation analysis step, it will work with another set of images or sequences that we call the “*testing set.*” For the “testing set,” the DMOS values are unknown; therefore, we obtain them via (1).

In our study, all the metrics have been “trained” only with the luminance information. The MSSIM, VIF, RRIQA, and DMOSp-PSNR metrics were “trained” with the whole Live2 Database because they are intended to be generalist metrics.

The NRJPEGQS was “trained” only with the JPEG distorted images of Live2 database as this metric is designed only to handle this type of distortions. And for the same reason the NRJPEG2000 was “trained” only with the JP2 K distorted images of Live2 Database and the VQM-GM was “trained” with a subset of 8 video sequences and its 9 corresponding HRCs of the VQEG Phase I Database in a bitrate range of 1 to 4 Mb/s.

It is important to mention that each of these “training sets” has different dynamic ranges in the DMOS scale depending on the degree of distortions applied to the images in each set.

We define as “homogeneous metrics” those which were trained with the same sets, and therefore, we use the term “heterogeneous metrics” to refer to metrics that were trained with different sets.

Our “testing set” comprises different standard video sequences that are commonly used in video coding evaluation research, as shown in Table 3. For FR-metrics, both reference and distorted images/sequences are used as input. For NR-metrics only the distorted image/sequence is available. For RR-metrics, the reference image/sequence is the input of the features extraction step, and both the extracted features and the distorted image/sequence are the input for the final metric evaluation step. Image metrics were applied to each frame of the sequences and the mean raw value for all the frames was translated to the DMOSp scale. Hence, we finally obtain comparable DMOSp values for all images/sequences.

## 4. Analyzing Metrics Behavior in a Compression Environment 

In this section, we will study the behavior of the QAM under evaluation when assessing the quality of compressed images and sequences with different encoders. As exposed before, in the development of a new encoder or when performing modifications to existing ones, the performance of the proposals must be evaluated in terms of perceived quality by means of the R/D behavior of each encoder. The distortion metric commonly used in the R/D comparisons is PSNR.

So, in this test environment, we will work with the selected metrics as candidates to replace the PSNR as the quality metric in a R/D comparison of different video codecs. In this case, we will use a set of video encoders and video sequences in order to create distorted sequences hypothetical reference circuit (HRC) at different bitrates and analyze the results of the different QAM under study. Also, we will consider the metric complexity in order to determine their scope of application. For the tests, we have used an Intel Pentium 4 CPU Dual Core 3.00 GHz with 1 Gbyte RAM. The programming environment used is Matlab 6.5 Rel.13. The codecs under test are H.264/AVC [65], Motion-JPEG2000 [66], and Motion-LTW [67]. The fitting between objective metric values and subjective DMOS scores was done using the Matlab curve fitting toolbox looking for the best fit in each case.

**Figure 5:**
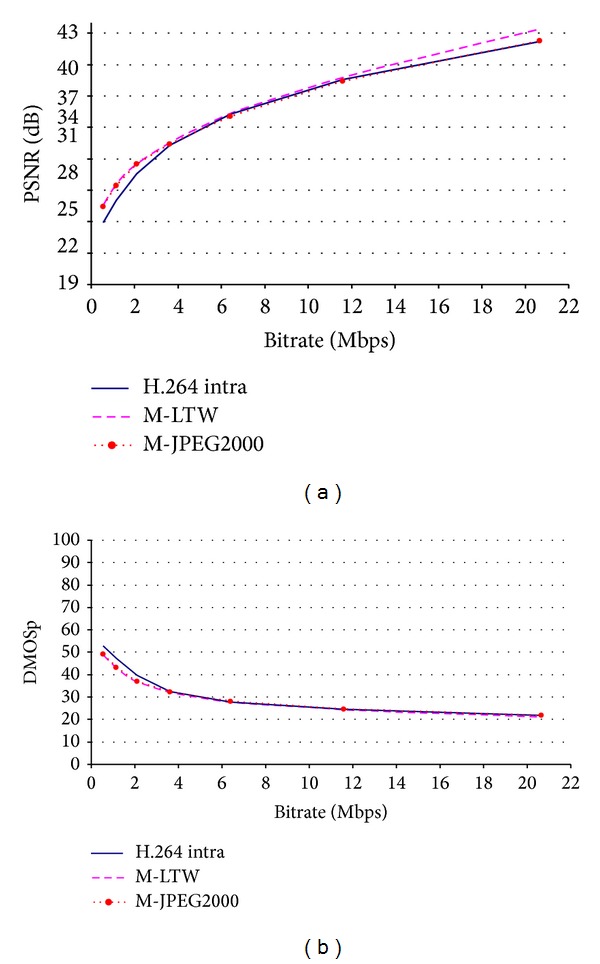
PSNR versus DMOSp-PSNR for the evaluated codecs (mobile sequence).

A R/D plot of the different video codecs under test, using the traditional PSNR as a distortion measure, is shown in Figure 5(a). It is usual to evaluate performance of video codecs in a PSNR range varying from 25–27 dB to 38–40 dB, because it is difficult to determine which one is better with PSNR values above 40 dB. This saturation effect, at high qualities, is not captured by the traditional PSNR that increases steadily as the bitrate rises, as shown in Figure 5(a).

We convert the traditional PSNR to a metric that we call DMOSp-PSNR by applying the scale-conversion process explained in Section 3. We can consider the DMOSp-PSNR metric to be the “*subjective*” counterpart of the traditional PSNR. It is the same metric, though expressed in a different scale. The DMOSp scale denotes distortion, thereby quality increases as DMOSp value decreases. The main difference between PSNR and its counterpart DMOSp-PSNR is that the saturation effect is fixed, as we can see in Figure 5(b). As it can be seen, subjective saturation effect is noticeable above a specific quality value. At bitrates above 11.5 Mbps, the DMOSp values practically do not change. This behavior is the same for all the evaluated codecs and video formats, confirming that there is no noticeable subjective difference when watching the sequences at the two highest evaluated bitrates (11.7 and 20.7 Mbps).

But as mentioned before the only modification that has been done to the PSNR metric was the mapping process with the DMOS data; that is, the raw values of the PSNR have not changed; therefore, DMOSp-PSNR metric does not fix the known drawbacks shown in Figure 1. For bitrates values below the saturation point (11.5 Mbps in the case of Figure 5(b)), the behavior of the two R/D curves is the same. In fact, the DMOSp-PSNR metric below the saturation point arranges the codecs by quality in the same order as the PSNR does, agreeing also with the results of subjective tests. This behavior is the same for all evaluated sequences and bitrates.

Since PSNR, and therefore DMOSp-PSNR, are known to be inaccurate perceptual metrics for image or video quality assessment, we now analyze the remaining metrics under study for all codecs and bitrates. These metrics have a better perceptual behavior and they offer different scores for the images in Figure 1.

The expected behavior of a QAM scoring an image or sequence at different bitrates is as follows.

- It should give a decreasing quality value as the bitrate decreases when bitrate values are below saturation threshold.
- The quality value should be almost the same when bitrate values are above saturation threshold.

So, we run all the metrics for each HRC and analyzed the resulting data between consecutive bitrates, obtaining the quality scores in the DMOSp space. A simple subjective DSCQS test was performed with 23 viewers in order to detect if there was really perceived differences above threshold in these sequences at high bitrates (above saturation 11.5 Mbps). In the tests, the three HRCs (for each sequence and encoder) with higher bitrates were presented to the viewers: the first HRC (the first located below saturation point, 6.4 Mbps) and the last two HRCs (two rightmost points from curves in Figure 5, 11.58 and 20.65 Mbps) that are locate in the saturation region. The test concluded that no perceptual differences were detected above saturation threshold, whereas all the viewers detected some perceptual differences bellow threshold. The predicted DMOSp differences for these HRCs above threshold vary from 0.82 to 4.91 DMOSp points, so we can initially conclude that above saturation these small differences in DMOSp values are perceptually indistinguishable.

**Figure 6:**
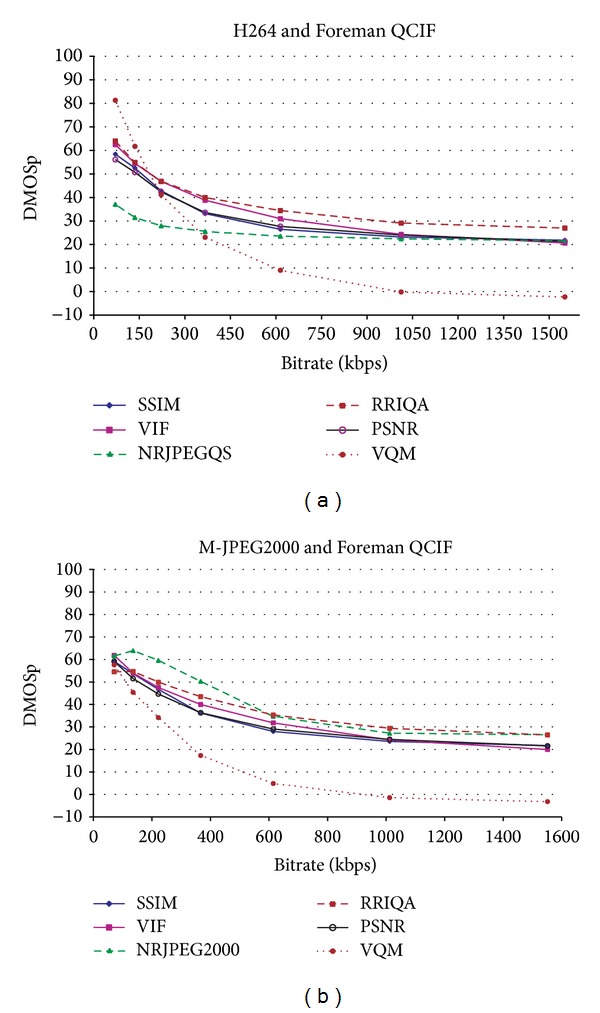
QAM comparison using the same sequences with different codecs.

In Figure 6 we can see examples of the R/D plots used for comparing the metrics where all the evaluated QAM were applied to the same sequence. In Figure 6(a), the HRCs were encoded with the H.264/AVC codec. The NRJPEG2000 metric is omitted because it is not designed to handle DCT transform distortions. In the same way, in Figure 6(b), where HRCs were encoded with M-JPEG2000, the NRJPEGQS metric is omitted because it is not designed to handle the distortions related to the wavelet transform. We can see that the perceptual saturation is captured by all the QAM at high bitrates (high quality) regardless of the encoder. The same holds for all the sequences and encoders.

**Figure 7:**
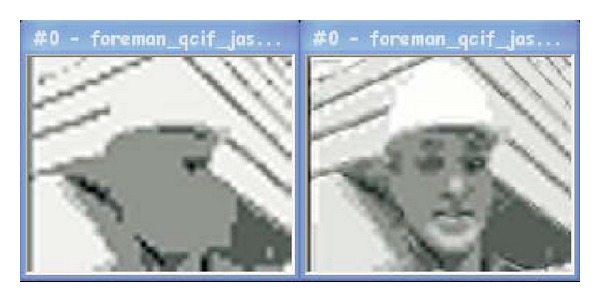
First frame of Foreman QCIF encoded at 70 Kbps (left) and 135 Kbps (right).

As mentioned in Section 3, monotonicity is expected in the mapping function. So, the expected behavior of the metrics should also be monotonic; that is, metrics should indicate lower quality values as the bitrates decrease. However, if we look at Figure 6(b) and focusing on the two lowest bitrates, the quality score given by both the RRIQA and NRJPEG2000 metrics increases as the bitrate value decreases. This is contrary to the expected behavior of a QAM.  Figure 7 shows the first frame of the Foreman QCIF frame size sequence at these bitrates. Clearly, the right image (135 Kbps) receives a better subjective score than the left one (70 Kbps), though the mentioned metrics state just the opposite in this particular case. Our results for the compression environment show that NRJPEG2000 offers wrong quality scores between the two highest compression ratios with the M-JPEG2000 codec, for all the sequences and frame sizes tested. RRIQA also failed with this codec at high compression ratios, but only for small video formats. All the other metrics exhibit a monotonic behavior for all bitrates regardless of the encoder and sequence being tested.

Figure 6 will also help us to explain what it was exposed in Section 3; heterogeneous metrics should not be compared directly because the dynamic range of the subjective quality scores in each training set is different. Looking at Figure 6(a) and focusing on the lowest bitrate, the DMOSp rating differences between metrics arrive surprisingly up to 44.21 DMOSp units.

In fact, there are three different behaviors corresponding to the use of three different training sets: VQM-GM was trained with VQEG sequences, NRJPEGQS was trained only with the JPEG distorted images, and the rest of the metrics trained with the whole set of distorted images in the Live2 Database. This is the main reason of these anomalous behaviors in Figure 6.

So, when including in the same R/D plot curves from different metrics it should be checked that the metrics are homogeneous in order to avoid misleading conclusions.

Determining how good a metric works depends on how good the metric predicts the subjective scores given by human viewers. This goodness of fit is measured in parameters like those of Table 2. Our performance validation data tells that the VIF metric is the one which best fits the subjective DMOS values among the metrics in the same “training set.”

**Figure 8:**
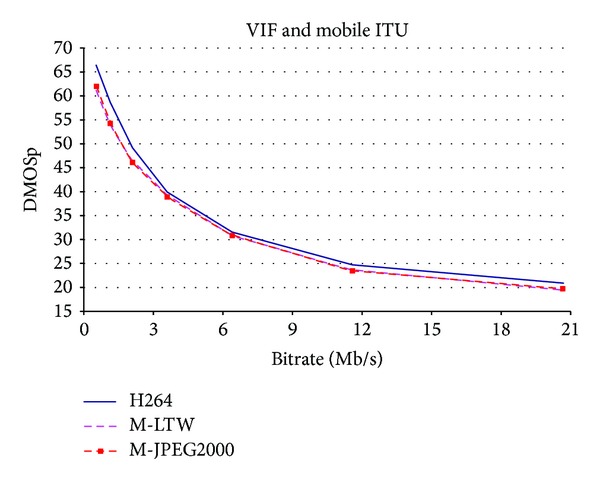
R/D performance evaluation of the three video codecs using mobile ITU video sequence by means of VIF metric.

Figure 8 represents the common R/D plots used when comparing the performance of the encoders being tested. In this case the plot shows how the VIF metric evaluates the performance of the encoders. If the mapping function of the metrics was obtained with the same “training set,” then the ranking order of the encoders should agree with the subjective ranking order for each bitrate being evaluated.

We performed a simple subjective test with 23 viewers in order to evaluate if we can trust the codec ranking; that is, for a specific bitrate, the metric should arrange the encoders by quality, in the same order that a human observer does. For each rate and sequence, the reconstructed sequence of each encoder was presented simultaneously to the subjects. The ordering of the three sequences varies for each HRC, so that the subjects had no knowledge about the encoder order. The subjects ranked the sequences by perceptual quality if no differences were detected between pairs of sequences; they also annotated this fact. After analyzing the users scores and removing outliers, the test confirms that the ranking order of the metrics was the same as the subjective ranking.

In the cases where viewers scored no perceptual difference between sequences, the metrics gave always values lower than 2.9 DMOSp units of difference between encoders. In this test, for slightly higher differences, for example, 3.11 DMOSp units at 2.1 Mb/s between H264/AVC and M-JPEG2000 in Figure 8, most of the viewers could see some perceptual differences between the sequences, since they ranked H264/AVC to have better perceptual quality than M-JPEG2000 and M-LTW.

In order to determine how much difference expressed in the DMOSp scale is perceptually detectable, deeper studies and subjective tests must be done. From our studies, we detect that the perceptual meaning of the difference depends on the point in the DMOSp scale where we are working. For example, for high quality (as stated before in previous tests), DMOSp value differences up to 4.91 DMOSp points were imperceptible; however, at lower quality levels, smaller differences (3.11) can be perceived.

Finally, Table 4 shows, for different frame sizes, the mean frame evaluation time and the evaluation time for the whole sequence needed by each metric to assess its raw quality value. Times for the two steps of RRIQA, features extraction (f.e.), and quality evaluation (eval.) have been separately measured. For a CIF sequence (calibration and colour conversion time is not included) the VQM-GM is faster than the other metrics, except NRJPEGQS and DMOSp-PSNR. DMOSp-PSNR is by far the less computationally expensive metric at all frame sizes. On the other hand, RRIQA and VIF are the slowest metrics (they run a linear multiscale, multiorientation image decomposition), although in our tests the VIF is the most accurate metric among the general purpose metrics.

## 5. Analyzing Metrics Behaviour in a Packet Loss Environment

Our objective in this section is to analyze the behavior of the candidate metrics in the presence of packet losses under different MANET scenarios. In order to model the packet losses in these error prone scenarios, we use a three-state hidden Markov model (HMM) and the methodology presented in [68]. HMMs are well known for their effectiveness in modeling bursty behavior, relatively easy configuration, quick execution times, and general applicability. So, we consider that they fit our purpose of accelerating the evaluation process of QAM for video delivery applications on MANET scenarios, while offering similar results to the ones obtained by means of simulation or real-life testbeds. Basically, by the use of the HMM, we define a packet loss model for MANET that accurately reproduces the packet losses occurring during a video delivery session.

The modeled MANET scenario is composed of 50 nodes moving in an 870 × 870 square meters area. Node mobility is based on the random way-point model, and speed is fixed at a constant value between 1 and 4 m/s. The routing protocol used is DSR. Every node is equipped with an IEEE 802.11g/e enabled interface, transmitting at the maximum rate of 54 Mbit/s up to a range of 250 meters. Notice that a QoS differentiated service is provided by IEEE 802.11e [69]. Concerning traffic, we have six sources of background traffic transmitting FTP/TCP traffic in the best effort MAC access category. The foreground traffic is composed by real traces of an H.264 video encoded (using the Foreman CIF video test sequence) at a target rate of 1 Mbit/s. The video source is mapped to the video MAC acess category.

We apply the HMM described above to extract packet arrival/loss patterns for the simulation traces and later replicate these patterns for testing. We describe two environments: (a) congestion related environment and (b) mobility related environment.

The congestion environment is composed of 6 scenarios with increasing level of congestion, from 1 to 6 video sources. The mobility environment is composed of 3 scenarios with only one video source, but with increasing degrees of node mobility (from 1 to 4 m/s).

For each of these scenarios, we get different packet loss patterns provided by the HMM that represents each scenario.

After an analysis of the packet losses, different patterns are defined as follows.

- Isolated small bursts represent less than 7 consecutive lost packets. As each frame is split in 7 packets at source, isolated bursts will affect 1 or 2 frames, but none of them will be completely lost. This error pattern is mainly due to network congestion scenarios, where some packets are discarded due to transitory high occupancy in the wireless channel or buffers at relaying nodes.
- Large packet loss bursts. Large Bursts cause the loss of one or more consecutive frames. Large packet error bursts are typically a consequence of high mobility scenarios, where the route to the destination node is lost and a new route discovery process should be started. This will keep the network link in down state during several seconds, losing a large number of consecutive packets.

We have used the H.264/AVC codec adjusting the error resilience parameters to the values proposed in [70], so that the decoder is able to reconstruct sequences even when large packet loss bursts occur. H.264/AVC is configured to produce one I frame every 29 P frames, with no B frames and to split each frame in 7 slices, so we put each slice into a separate packet and encapsulate its output in RTP packets. As suggested in [70], we also force 1/3 of the macroblocks of each frame to be randomly encoded in intramode.

We have used the Foreman CIF seq. (300 frames at 30 fps) to build an extended video sequence by repeating the original one up to the desired video length. After running the encoder for each extended video sequence, we get RTP packet streams. Then, we delete from the RTP packet stream, those packets that have been marked as lost packets by the HMM model. This process simulates packet losses in the MANET scenarios, so a distorted bitstream will be delivered to the decoder. The decoder behavior depends on the packet loss burst type as follows.

- When an isolated small bursts appear, the decoder is able to apply error concealment mechanisms to repair the affected frames. The video quality decreases, and just after the burst, the reconstructed video quality recovers the quality by means of the random intracoded macroblock updating. When the next I frame arrives, it completely stops error propagation.
- When the decoder faces large bursts, it stops decoding and waits until new packets arrive. This produces a sequence in the decoder that is shorter than the original one. Therefore, both sequences are not directly comparable with the QAM and so we freeze the last completely decoded frame until the burst ends.

Once we have comparable video sequences (original and decoded video sequences with the same length), we are able to run the QAM. Each metric produces an objective quality value for each frame in its own scale. Then, we perform the scale-conversion to the DMOSp scale (see Section 3).

**Figure 9:**
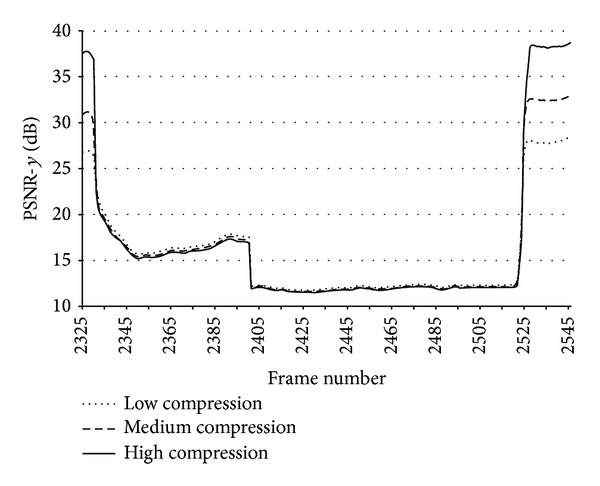
PSNR frame values during a long packet loss burst (from frame 2327 to 2525) at different bitrates.

Figure 9 shows the objective quality value in the traditional PSNR scale at three different compression levels (low compression, medium compression, and high compression) during a large packet loss burst. We observe the evolution of quality during the burst period. What the observer sees during this large burst is a frozen frame, with more or less quality depending on the compression level. The PSNR metric reports that quality drops drastically with the first frame affected by the burst and decrease even more as the difference between the frozen frame and the current frame increases. Nearly at the middle of the burst, an additional drop of quality can be observed. It corresponds to a scene change (with the beginning of a new cycle of the foreman video sequence). At this point, the drastic scene change makes the differences between sequences even higher, and the PSNR metric scores with even worse values, reaching values as low as 10–12 dBs.

On the other hand, the perceived quality which changes at these levels is quite difficult to evaluate. So, a better perceptually designed QAM should not score such a quality drop in this situation because quality saturates. When the burst ends, quality rapidly increases because of the arrival of packets belonging to the same frame number than the current one in the original sequence (frame 2525 in Figure 9).

If during such a burst a QAM takes into account only the quality of the frozen frame, disregarding the differences with the original one (which changes over time), the effect of the burst would remain unnoticed for that metric, that is, quality remain constant.

**Figure 10:**
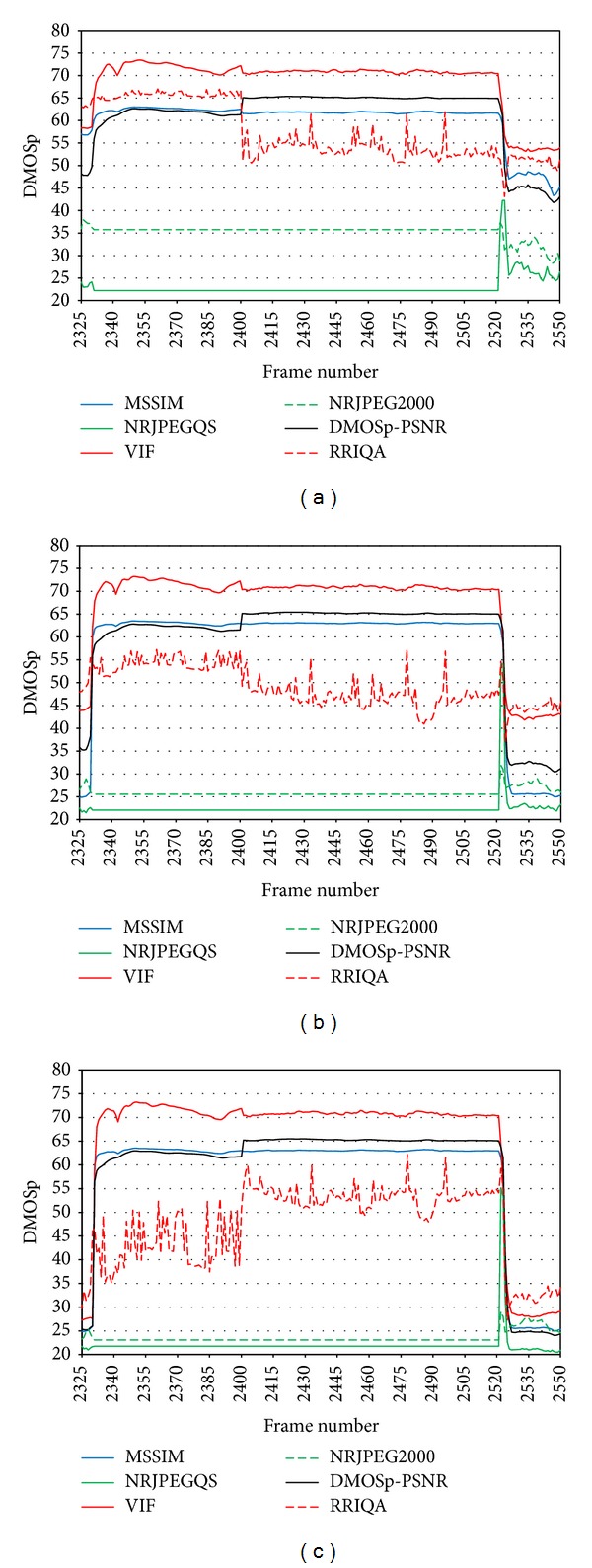
Metric comparison in the DMOSp space during a very large burst.

Figure 10 shows the evolution of the candidate QAM during a large burst (similar to Figure 9 but in this case in the DMOSp space). There is a panel for each compression level: Figure 10(a) corresponds to high compression, Figure 10(b) to middle compression, and Figure 10(c) to low compression. We observe some interesting behaviors that we proceed to analyze.

From a perceptual point of view, quality must drop to a minimum when one or more frames are lost completely and should remain that way until the data flow is recovered. It should not matter if a scene change takes place inside the large burst. VIF and MSSIM behaves this way. At the point of the burst, where the scene change takes place, both the VIF and MSSIM metrics have almost reached their “bad quality” threshold regardless of the compression level and therefore there is no substantial change in the reported quality. The drop of quality to the minimum at the beginning of the burst evidence the lost of whole frames.

**Figure 11:**
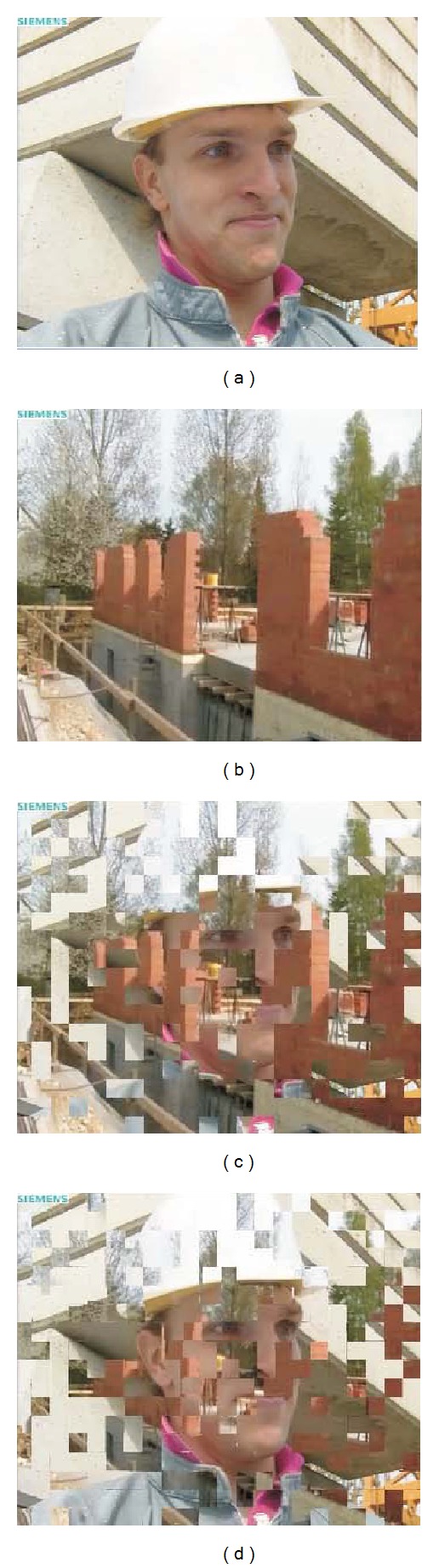
Frame reconstruction after a large burst: (a) original frame, (b) last frozen frame, and (c) (d) first and second reconstructed frames after the burst.

NR metrics do not detect the presence of a frozen frame (by dropping the quality score) as expected because the quality given by these metrics remain at the level scored for the frozen frame during the burst duration. So, NR metrics could not detect the beginning of a large burst, since lost frames will be replaced with the last correctly decoded frame (frozen frame) and the reference frames are not available for comparison. However, NR metrics detect the end of such bursts.  Figure 11 will help us to explain this behavior, showing how reconstruction is done after a large burst. This figure shows the impairments produced when the large burst ends.  Figure 11(a) is the current frame, the one being transmitted.  Figure 11(b) is the frozen frame that was repeated during the burst duration. When the burst ends, the decoder progressively reconstruct the sequence using the intramacroblocks from the incoming video packets. So the decoder partially updates the frozen frame with the incoming intramacroblocks. This is shown in Figures 11(c) and 11(d) where the face of the foreman appears gradually.

**Figure 12:**
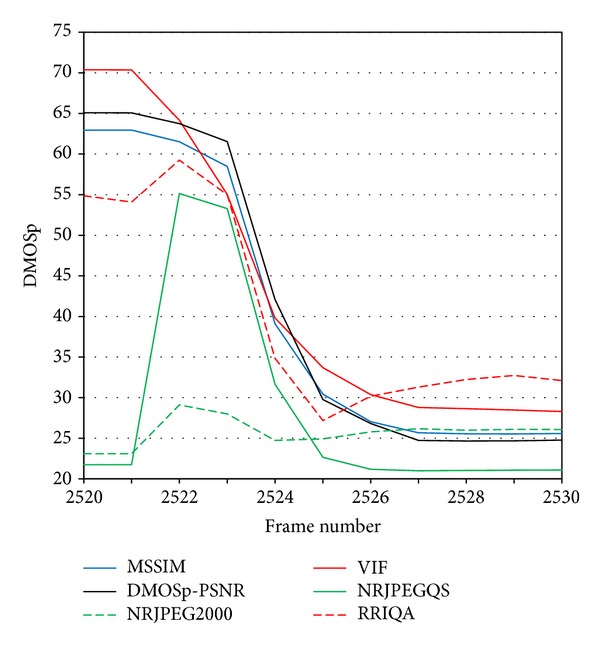
End of the large burst for the low compression panel. FR and NR metrics show the opposite behavior.

The gradual reconstruction of the frame with the incoming macroblocks is interpreted in a different way by NR metrics and FR metrics. When the macroblocks begin to arrive, what happens at frame 2522 (see Figure 12), the NR metrics react scoring down quality, while the FR metrics begin to increase their quality score, just the opposite behavior. For a NR metric, without a reference frame, Figure 11(c) has clearly worse quality than Figure 11(b). But for a FR metric the corresponding macroblocks between Figures 11(c) and 11(a) help to increase the scored quality.

So, NR metrics react only when the burst of lost packets affects frames partially, that is, isolated bursts and at the end of a large burst. The NRJPEGQS metric reacts harder (i.e., it shows higher quality differences) than the NRJPEG2000 because it was designed to detect the blockiness introduced by the discrete cosine transform. When the frame is fully reconstructed then the score obtained with NR and FR metrics approaches again the values achieved before the burst, which depends on the compression rate.

The RRIQA metric shows high variability in its scores between consecutive frames inside bursts. These variations become more evident as the degree of compression decreases. The nature of the data sent through the ancillary channel, 18 scalar parameters obtained form the histogram of the wavelet subbands of the reference image, is very sensitive to loss of synchronism between the reference frame and the frozen one. On the decoder, the same extracted parameters are statistically compared with the received through the ancillary channel. When this comparison is performed with two sets of parameters obtained from different frames, unexpected results appear.

Concerning the FR metrics, MSSIM, VIF, and PSNR-DMOSp show a similar behavior or trend. MSSIM and PSNR-DMOSp show closer quality scores between them than the ones obtained with the VIF metric, which gives lower quality values than the other two metrics. This behavior is the same regardless of the compression level inside the large burst. Leaving aside the PSNR-DMOSp, which is not really a QAM, the other two FR metrics (VIF and MSSIM) have the same behavior when facing large bursts.

**Figure 13:**
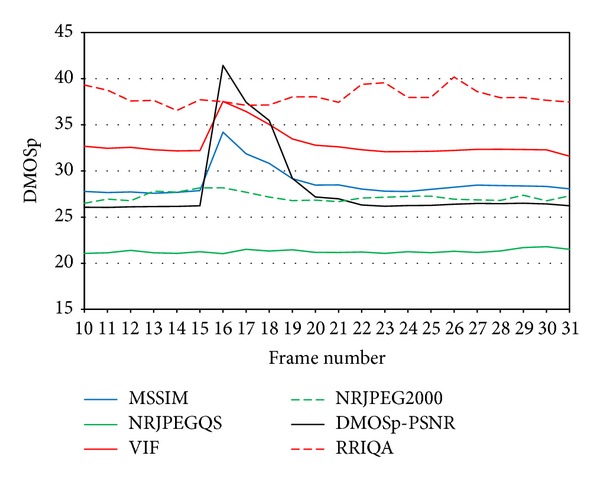
Metric comparison for an isolated burst.

**Figure 14:**
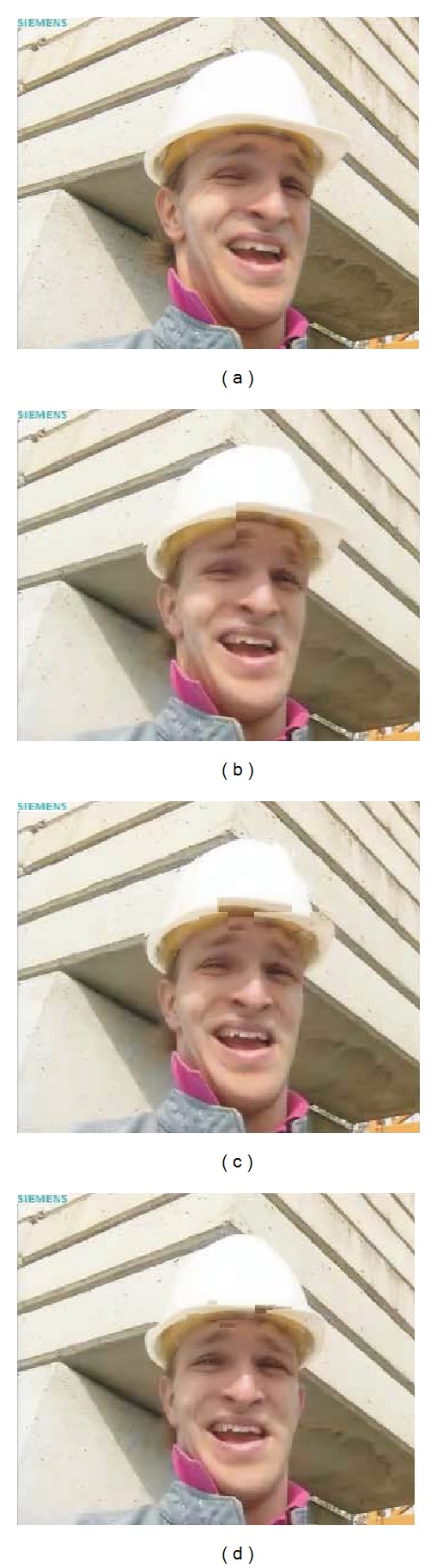
Packet loss affecting only one frame. (a) Original frame and (b, c, d) next three decoded frames.

Figure 13 shows an isolated burst. In this case, blur and edge shifting impairments are introduced altering only one frame. This fact is perceived only by the FR metrics and the NRJPEG2000, which is designed to detect this type of impairments. The error concealment mechanism of H.264/AVC needs up to 6 frames to achieve the same quality scores obtained before the burst.  Figure 14 shows the original frame (a) and three subsequent frames (b, c, d), where the effect of the lost packets is concealed by the H.264/AVC decoder.

As defined previously, an isolated burst can affect one or two consecutive frames. In the last case, the behavior of the QAM when facing the isolated burst resembles the behavior of the metrics with a large burst. The difference is that the concealment mechanisms and the correct reception of part of the frames avoid the largest drop in the quality.

**Figure 15:**
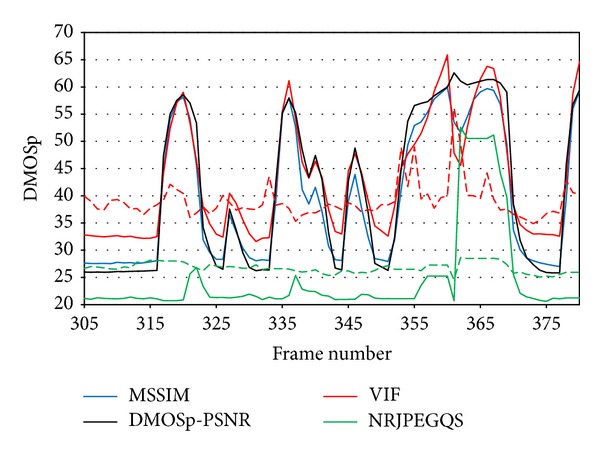
Frame interval where different type of bursts occurs consecutively.

Figure 15 shows multiple consecutive bursts (large and isolated) that behave as exposed previously. From left to right, we see a large burst followed by an isolated one. This pattern repeats again one more time, and at the right most part of the figure, between frames 352 and 372, two large bursts occur consecutively, having a gap between them where new incoming packets arrive for a short period of time (frames 361 and 362).

**Figure 16:**
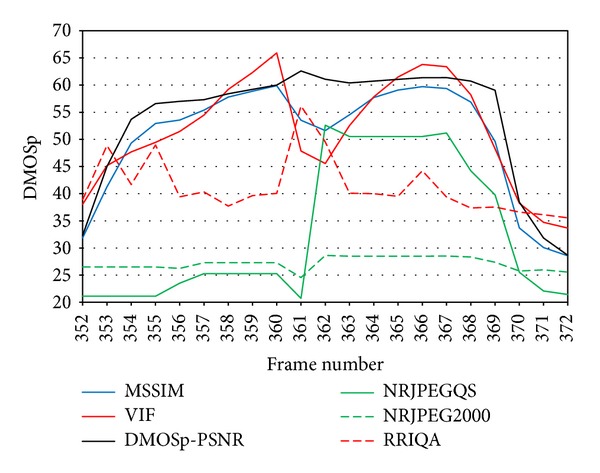
Detail from two consecutive long burst with incoming packets between them.

**Figure 17:**
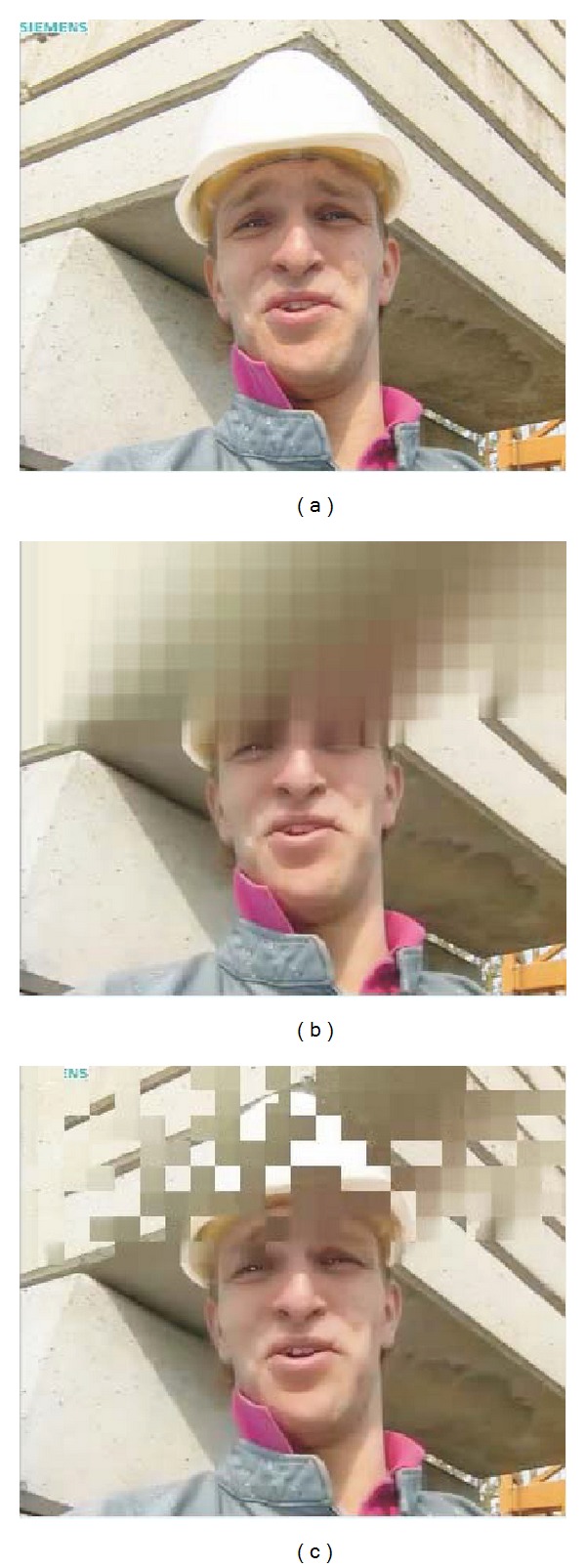
Decoded frames between two consecutive bursts: (a) original frame; reconstructed frames (b) 361 and (c) 362.

In Figure 16, we zoom into this area (frames 352 to 372) to analyze why the behavior of the DMOSp-PSNR metric differs from the other FR metrics during the gap between bursts. In the gap, the encoder is not able to reconstruct a whole frame because the gap is too small, that is, between the two large bursts only a small amount of packets arrive, and this is not enough to reconstruct a whole frame. So the involved frames (361 and 362) are partially reconstructed (Figures 17(b) and 17(c)). Both frames exhibit perfect correspondence in the lower half with the original one (Figure 17(a)). Therefore, the scored quality must increase, at least to some extent, compared to the quality of the previous frozen frame, as occurs at the end of a large burst. This fact is only reflected by the VIF and MSSIM metrics. The PSNR-DMOSp metric is not able to detect this because it is computed using information from the whole frame. For the VIF and the MSSIM, which are perceptually driven, the lower half of the frame increases their raw scores, in the same way as the human scores do. After frame 362, quality decreases again since the following frame is frozen too. So, VIF and MSSIM detect two consecutive loss burst, while PSNR-DMOSp and the other metrics consider only a single larger one.

## 6. Conclusions

The main goal of this work was focused on looking for a quality assessment metric that could be used instead of the PSNR when evaluating compressed video sequences with different encoder proposals at different bitrates and to analyze the behavior of such metrics when compressed video is transmitted over error prone networks such as MANETs.

We explained the procedures that we followed to compare QAM metrics and alerted about some issues that arise when a comparison between heterogeneous metrics is made. The metrics must be compared using a common scale, since the raw scores of the metrics are not directly comparable. The scale-conversion process involves subjective tests and the use of mapping functions between the subjective MOS values and the metrics raw values. The parameters for the mapping function we used are provided in the paper. The metrics were first trained with a set of images from two open source image and video databases with known MOS values. The metrics were tested with another set of images and videos also taken from available databases. In order to perform a fair comparison, the training and testing sets used with each metric must use only impairments which the metric was designed to handle. We defined as heterogeneous metrics those that were trained with different sets of images or sequences. The R/D comparisons of heterogeneous metrics must be done carefully, focusing not only on the absolute quality scores, but also on the relative scoring between consecutive bitrates as the differences between DMOSp values are perceptually detected (or not) depending on the quality range. When metrics are trained with the same training set, differences in DMOSp values have the same perceptual meaning for all the metrics, but this may not be true between heterogeneous metrics. Normalizing the DMOSp scale when comparing heterogeneous metrics helps to detect these differences.

We performed the comparison between metrics in two environments: a compression environment and a packet loss environment. We performed several subjective tests in order to confirm that the analysis and the behavior of the metrics were consistent with human perception. Our tests included the comparisons of three encoders by replacing the PSNR as distortion metric in their R/D curves with each of the candidate metrics.

From our results of the compression environment, we conclude that we can trust the quality provided by the VIF metric, which is the one that obtains a better fit in terms of DMOS during the calibration process and on how it ranks the performance of the tested encoders for the bitrate range under consideration. The NRJPEG2000 and the RRIQA metrics break monotonicity for very high compression levels when M-JPEG2000 is the evaluated encoder. For the rest of the bitrates, all the other metrics show a monotonic behavior for all the bitrate range and for all encoders.

The choice of a QAM to replace the traditional PSNR, when working in a compression framework with no packet losses, depends on the availability of the reference sequence. In applications where the reference sequence is not available, RRIQA is our choice because its behavior is similar to FR metrics. If the reference sequence is available, the choice depends on the weight given to the tradeoff between computational cost and accuracy. If time is the most important parameter, we will choose DMOSp-PSNR followed by VQM and MSSIM. If accuracy is more important, then the choice will be VIF and MSSIM metrics.

In the loss-prone environment, we analyzed the metrics behavior when measuring reconstructed video sequences encoded and delivered through error prone wireless networks, like MANETs. In order to obtain an accurate representation of delivery errors in MANETs, we adopted an HMM model able to represent different MANET scenarios.

The results of our analysis are as follows. (a) NR metrics are not able to properly detect and measure the sharp quality drop due to the loss of several consecutive frames. (b) The RR metric has a nondeterministic behavior in the presence of packet losses, having difficulties in identifying and measuring this effect when the video is encoded with moderate to high compression rates. (c) Concerning the other metrics, MSSIM, DMOSp-PSNR, and VIF show a similar behavior in all cases. In summary, we consider that although they exhibit slight differences in the packet loss framework, we propose the use of the MSSIM metric as a tradeoff between a high quality measurement process (resembling human visual perception) and computational cost.

## Figures and Tables

**Table 1:** Equation parameters of metrics under study.

	*β*_1_	*β*_2_	*β*_3_	*β*_4_	*β*_5_
MSSIM	−39.5158	14.9435	0.8684	−10.8913	46.4555
VIF	−3607.3040	−0.5197	−1.6034	−476.0144	−693.3585
NRJPEGQS	37.6531	−0.9171	6.6930	−0.2354	40.7253
NRJPEG2000	37.3923	0.8190	0.6011	−0.8882	74.5031
RRIQA	−18.9995	1.5041	3.0368	6.4301	5.0446
PSNR-DMOSp	23.2897	−0.4282	28.7096	−0.6657	61.5160
VQM-GM	−163.6308	6.3746	−7.6192	114.4685	76.6525

**Table 2:** Goodness of DMOSp-DMOS fitting.

	PCC	RMSE	SROCC
MSSIM	0.8625	7.9682	0.851
VIF	0.9529	0.0516	0.9528
NRJPEGQS	0.936	3.0837	0.902
NRJPEG2000	0.9099	7.056	0.9021
RRIQA	0.9175	4.4986	0.9194
PSNR-DMOSp	0.85257	9.0969	0.8197
VQM-GM	0.8957	7.6746	0.9021

**Table 3:** Sequences included in the “test set”.

Sequence	Frame	F. number	F. rate
Foreman	QCIF: 176 × 144	300	30 fps.
Container			
Foreman	CIF: 352 × 288		
Container			
Mobile	640 × 512	40	

**Table 4:** QAM average scoring times (seconds) at frame and sequence level.

	QCIF		CIF		640 × 512	
	Frame	Seq.	Frame	Seq.	Frame	Seq.
MSSIM	0.028	8.4	0.147	44.1	0.764	30.5
VIF	0.347	104.1	1.522	456.5	6.198	247.9
NRJPEGQS	0.01	3	0.049	14.6	0.201	8.1
NRJPEG2000	0.163	48.9	0.486	145.9	1.595	63.8
RRIQA (f.e.)	4.779	1433.7	6.95	2084.9	10.111	404.5
RRIQA (eval.)	0.201	60.2	0.635	190.6	2.535	101.4
DMOSp-PSNR	0.001	0.3	0.006	1.7	0.02	0.8
